# Deltamethrin-Mediated Effects on Locomotion, Respiration, Feeding, and Histological Changes in the Midgut of *Spodoptera frugiperda* Caterpillars

**DOI:** 10.3390/insects12060483

**Published:** 2021-05-22

**Authors:** Germano Lopes Vinha, Angelica Plata-Rueda, Marcus Alvarenga Soares, José Cola Zanuncio, José Eduardo Serrão, Luis Carlos Martínez

**Affiliations:** 1 Department of Crop Science, Federal University of Viçosa, Viçosa 36570000, Brazil; germano.lopes.vinha@gmail.com; 2 Department of Entomology, Federal University of Viçosa, Viçosa 36570000, Brazil; plata.angelica@gmail.com (A.P.-R.); zanuncio@ufv.br (J.C.Z.); 3 Department of Crop Production, Federal University of Vales Jequitinhonha and Mucuri, Diamantina 39100000, Brazil; marcus.alvarenga@ufvjm.edu.br; 4 Department of General Biology, Federal University of Viçosa, Viçosa 36570000, Brazil; jeserrao@ufv.br

**Keywords:** anti-feeding effect, histopathology, repellency, respiration rate, survival, toxicity

## Abstract

**Simple Summary:**

*Spodoptera frugiperda* is controlled mainly with chemical insecticides. Toxicity, survival, respiration, mobility, anti-feeding effect, and histology of the midgut of *S. frugiperda* caterpillars exposed to deltamethrin were evaluated. Deltamethrin was toxic to third-instar caterpillars, decreasing survival. The insecticide reduces the respiratory rate and food consumption, and causes repellency. Exposure to deltamethrin causes histological alterations in the midgut, damaging the digestive cells and peritrophic matrix. Deltamethrin is toxic to *S. frugiperda* caterpillars, causing mortality, alteration of locomotor behavior, reduced respiration and feeding, and irreversible damage to the midgut epithelium.

**Abstract:**

*Spodoptera frugiperda* (J.E. Smith) (Lepidoptera: Noctuidae) is the main pest of maize crops, and effective methods for pest management are needed. The insecticidal efficacy of deltamethrin was evaluated against *S. frugiperda* for toxicity, survival, locomotion, anti-feeding, and histological changes in the midgut. Concentration–mortality bioassays confirmed that deltamethrin (LC_50_ = 3.58 mg mL^−1^) is toxic to *S. frugiperda* caterpillars. The survival rate was 99.7% in caterpillars not exposed to deltamethrin, decreasing to 50.3% in caterpillars exposed to LC_50_, and 0.1% in caterpillars treated with LC_90_. *Spodoptera frugiperda* demonstrated reduced mobility on deltamethrin-treated surfaces. Deltamethrin promoted a low respiration rate of *S. frugiperda* for up to 3 h after insecticide exposure, displaying immobilization and inhibiting food consumption. Deltamethrin induces histological alterations (e.g., disorganization of the striated border, cytoplasm vacuolization, and cell fragmentation) in the midgut, damaging the digestive cells and peritrophic matrix, affecting digestion and nutrient absorption.

## 1. Introduction

*Spodoptera frugiperda* (Smith, J.E.) (Lepidoptera: Noctuidae) is a polyphagous insect pest of crops in many parts on the world [1,2]. In Brazil, successive soybean, maize, and cotton crops are vulnerable to destruction by *S. frugiperda* [3]. For alternative pest control, biological control agents have been used [4], but with limitations to manage *S. frugiperda* [5]. Transgenic plants expressing proteins of *Bacillus thuringiensis* (*Bt*) have high specificity for target pests [6], but the occurrence of resistant populations of *S. frugiperda* has been reported in Argentina, Brazil, and the USA [7,8]. Additionally, pathogens such as *Baculovirus spodoptera* and *S. frugiperda* Nuclear Polyhedrosis Virus (SpfrNPV) have low virulence in addition to the occurrence of resistant populations [9].

In Brazil, insecticides are the main defense against *S. frugiperda*, with more than 150 active chemical substances used for its control [10], including carbamates, organochlorines, organophosphates, and pyrethroids [11]. In this scenario, deltamethrin, a neurotoxic insecticide of the pyrethroids group, acts on sodium channels in the plasma membrane of nerve cells [12,13]. This insecticide is used to control certain cotton [14], maize [15], oil palm [16], and urban pests [17]. The effect of deltamethrin on insects occurs both by contact and ingestion, and its effects were studied on Lepidoptera pests such as *Neoleucinodes elegantalis* Guenée (Crambidae) [18], *Cnaphalocrocis medinalis* Guenée (Pyralidae) [19], and *Tuta absoluta* Meyrick (Gelechiidae) [20].

In insects, several insecticides that act orally cause side-effects in the midgut [13,21,22]. The midgut, with functions such as nutrient absorption and digestive processes [23], cell regeneration [24], acting as a barrier against pathogens [25], and detoxification of chemical substances [26,27], is the first organ exposed to insecticides per os. Thus, insecticide molecules should cross that barrier to reach the hemolymph and spread to the target organ [28]. Therefore, the midgut is the major site of insecticide entrance in the insect body and toxic molecules first cause alterations in this organ to affect insect physiology [29,30,31].

Exposure to insecticides impacts negatively on non-target organs, which can change according to molecule and insect species [32]. Thus, the long-term effects of insecticides are poorly understood [33]. Deltamethrin has broad action of and toxicity against arthropods [34], being the major insecticide used to control maize pests such as *S. frugiperda* [35,36]. In this research, the action of deltamethrin on *S. frugiperda* survivorship, locomotion, respiration, feeding, and histotoxicity in the midgut were assessed.

## 2. Materials and Methods

### 2.1. Insects

A population of *Spodoptera frugiperda* (caterpillars and moths) was collected on non-*Bt* maize fields in Viçosa, Minas Gerais, Brazil, in December of 2018. The insects were maintained in the Laboratory of Biological Control of Insects of the Federal University of Viçosa on artificial diet for five generations using standard rearing techniques [37] with a population size above 500 insects to avoid inbreeding. Different developmental stages were reared at 28 ± 1 °C, 72 ± 15% relative humidity, and 12:12 h (light:dark) photoperiod. Adults of *S. frugiperda* were fed on a liquid diet (10% sucrose + 5% ascorbic acid solution + 85% water) in a moistened cotton ball. Every three days, papers with eggs were placed in plastic containers (1 L). Newly-hatched *S. frugiperda* caterpillars were kept in the plastic containers until the third-instar and subsequently individualized in PVC trays with 16 wells (Advento do Brasil, Diadema, SP, Brazil). These caterpillars were fed on a solid diet (31.2 g red beans, 12.5 g beer yeast, 25 g wheat germ, 25 g soybean protein, 12.5 g casein, 10 g agar, 1.2% ascorbic acid, 1.2% sorbic acid, 0.6% nipagin (methylparaben), distilled water, and 2.5 mL vitamin solution (0.015% niacin, 0.03% calcium pantothenate, 0.004% thiamine, 0.008% riboflavin, 0.004% pyridoxine, 0.008% folic acid, 0.002% biotin, 0.002% inositol, and 0.004% HCl)) ad libitum. Newly-emerged (24-h old) third-instar caterpillars of *S. frugiperda* with ca. 180 mg weight and without malformations were utilized in the bioassays.

### 2.2. Concentration–Mortality Bioassay

Deltamethrin (Decis^®^; 25 g L^−1^; Bayer Vapi Private Limited-Plot, São Paulo, Brazil) was diluted in 50 mL of distilled water for six concentrations (0.39, 0.78, 1.56, 3.12, 6.25, and 12.5 mg mL^−1^), where the maximum dilution was equivalent to 125 g a.i./L, corresponding with the field dose recommended for *S. frugiperda*. Water (distilled and deionized) was used in the control. Concentrations were prepared to evaluate the toxicity (acute or chronic) and determine the relevant toxicological endpoints, following the concentration–mortality relationship and lethal concentrations (LC_25_, LC_50_, LC_75_, and LC_90_) of this insecticide. Caterpillars were starved for 2 h before starting bioassay. Each solution (1 μL) was applied into 125 mg of artificial diet using an Eppendorf micropipette (1–10 μL, Eppendorf, Hamburg, Germany), which was supplied for the caterpillars during the first day. After this, for caterpillars feeding on the untreated or treated diet by 24 h, a new artificial diet without insecticide was supplied during the evaluation time of the experiment. Thirty third-instar caterpillars of *S. frugiperda* were individualized in Petri dishes (90 × 15 mm) and used for each concentration bioassay with three replications, following a completely random design. After five days of exposure, caterpillars were counted as dead if they were unable to walk when prodded with a brush.

### 2.3. Survival Bioassay

Caterpillars of *S. frugiperda* were individualized in Petri dishes and exposed to LCs (LC_25_, LC_50_, LC_75_, and LC_90_) of deltamethrin, determined by the concentration–mortality bioassay, in addition to the control with distilled and deionized water. Exposure procedures and insect conditions followed the same described for the concentration–mortality bioassay. Three replications of 30 caterpillars per lethal concentration were performed to determine the lethal time and the live insects recorded every 12 h for five days.

### 2.4. Locomotor Activity

*Spodoptera frugiperda* caterpillars were individually placed in a Petri dish 90 × 15 mm with a filter paper disc (Whatman No. 1) on the bottom (arena). The inner part of the top of the Petri dish was coated with Teflon^®^ PTFE (Dupont de Nemours Inc., Wilmington, DE, USA) to avoid insect escape. Behavioral response bioassays were performed in arenas half-treated with 250 µL of deltamethrin (LC_50_ or LC_90_), or control (distilled and deionized water). A caterpillar of *S. frugiperda* was released in the center of the arena half-treated with insecticide (in filter paper) and maintained for 10 min. Twenty-five caterpillars were used per treatment, and the experimental design was completely randomized. The locomotion of each insect in the arena was registered with a digital charge-coupled device (CCD) camera. The distance traveled and the resting time of *S. frugiperda* in each half of the arena was analyzed with a video-tracking system (ViewPoint Behavior Technology, Lyon, France). Insect was assumed as repelled or irritated when it spent < 60 s or 50% of the time in the half of the treated area with insecticide, respectively [38].

### 2.5. Respiration Rate

Bioassay was recorded for 3 h in *S. frugiperda* caterpillars after exposure to deltamethrin (LC_50_ or LC_90_), according to the procedure of the mortality–concentration bioassay and with those treated with water (distilled and deionized) used as a control. The production of carbon dioxide (CO_2_) (μL of CO_2_ h^−1^/insect) was measured with a respirometer of the type CO_2_ TR3C Analyzer (Sable System International, Las Vegas, EUA) [39]. Three *S. frugiperda* caterpillars were introduced into a 25 mL glass chamber in a completely closed system. The CO_2_ produced by one caterpillar was measured by 8 h at 26 ± 3 °C after insect acclimatization. The oxygen gas was injected through the glass chamber for 2 min at a flow of 150 mL min^−1^ to quantify the CO_2_ produced in the chamber. This airflow forced the CO_2_ molecules to pass through an infrared reader coupled to the system, allowing continuous measurement of the CO_2_ produced by insects in each chamber. *Spodoptera frugiperda* caterpillars, before and after the experiment, were weighed on an analytical scale (Sartorius BP 210D, Göttingen, Germany) and those with similar weight were evaluated. A total of fifteen caterpillars were used per insecticide (LC_50_ and LC_90_) concentration and control.

### 2.6. Anti-Feeding Effect

The application of the concentrations was carried out by the feeding method using maize leaves. Pieces of maize leaves (20 × 20 mm) were sterilized with 5% sodium hypochlorite, washed thrice with water (distilled and deionized), and dried at room temperature. Then, the pieces of maize leaf were soaked for 10 s in the LC_50_ and LC_90_ of deltamethrin and air dried for 1 h. *Spodoptera frugiperda* caterpillars were individualized in Petri dishes and fed on a piece of maize leaf treated with the insecticide or untreated (using distilled and deionized water as control). The caterpillar was in contact with maize leaf for 3 h and, subsequently, the piece was photographed with a Nikon D40 digital photographic camera (Nikon Corporation, Tokyo, Japan) with 15 cm macro focus, natural, and SB-700 Nikon flourishing light. The photos were analyzed with the QUANT v. 1.0 software (Federal University of Viçosa, Viçosa, MG, Brazil). The leaf area consumed by caterpillar was measured in mm² with pixels based on the RGB histogram (red, 763 nm; green, 581.6 nm; blue, 467.5 nm). A total of fifteen caterpillars were used for LCs (LC_50_ and LC_90_) of deltamethrin and control.

### 2.7. Histopathology

Ten third-instar caterpillars of *S. frugiperda* were exposed to LC_50_ of deltamethrin via ingestion, in addition to control (distilled and deionized water), for 3, 6, 12, and 24 h. The insects were anesthetized at −4 °C for 1 min, the midguts dissected in a saline solution for insects (0.1 M NaCl, 0.1 M KH2PO4, 0.1 M Na2HPO4), and fixed to Zamboni solution for 6 h at 4 °C. The midguts were dehydrated in a graded ethanol series (70, 80, 90 and 95%) and embedded in Leica historesin Leica (Leica Microsystems Inc., Buffalo Grove, IL, USA). Then, slices with 3 μm thickness were sectioned in a Leica RM2255 microtome, stained with H&S (hematoxylin–eosin), and observed under an Olympus BX-53 light microscope (Olympus Deutschland, Hamburg, Germany).

### 2.8. Statistical Analysis

The concentration–mortality curves were estimated by submitting the mortality data to Probit analysis, using the PROC PROBIT procedure with SAS v. 9.0 software. Time–mortality data were analyzed with the Kaplan–Meier survival analysis with Origin Pro v. 9.1 software. Data from the *S. frugiperda* caterpillars that remained alive at end of the experimental period were censored. The locomotor activity and anti-feeding effect were evaluated by analysis of variance (ANOVA) and means compared with Tukey’s (HSD; honestly significant difference) test (*p* < 00.5). A two-way ANOVA followed by Tukey’s HSD test (*p* < 00.5) were used to analyze the respiration rate data with treatments, time, and treatments×time interaction as fixed effects. Data analysis on locomotor activity, respiration rate, and anti-feeding effect were arcsine-transformed to satisfy assumptions of normality and homoscedasticity with the SAS v. 9.0 software.

## 3. Results

### 3.1. Concentration–Mortality Bioassay

Lethal toxicity results of deltamethrin on *S. frugiperda* caterpillars are shown in Table 1. From the Probit analysis, the LC_50_ was estimated at 3.58 mg mL^−1^ with a 95% confidence interval of 3.25–3.90 mg mL^−1^, whereas the LC_90_ of deltamethrin was 5.65 and 5.061–6.69 mg mL^−1^, respectively. Mortality in the control was <1%.

### 3.2. Survival Bioassay

**Figure 1:**
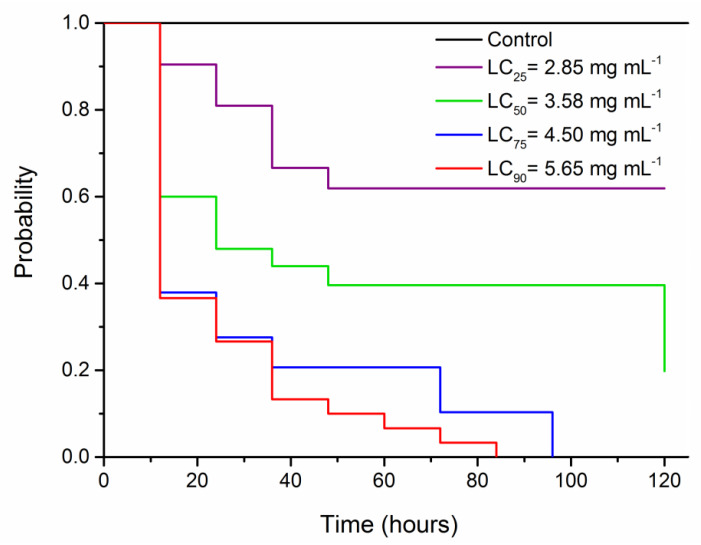
Survival curves of *Spodoptera frugiperda* caterpillars exposed to different lethal concentrations of deltamethrin, subjected to survival analyses using the Kaplan–Meier estimator log-rank test (χ^2^ = 64.55, df = 4, *p* < 0.001).

The survival analysis of *S. frugiperda* caterpillars exposed to the different LCs of deltamethrin indicated significant differences during the five days of evaluation (test of log-rank *χ*^2^ = 64.55, df = 4, *p* < 0.001). Survival was 99.7% in the control, decreasing to 71.8% with LC_25_, 50.3% with LC_50_, and 0.1% with LC_75_ and LC_90_ (Figure 1).

### 3.3. Locomotor Activity

**Figure 2:**
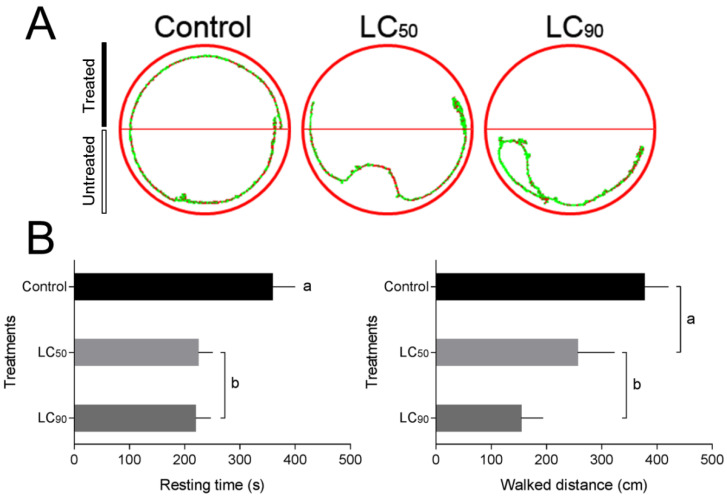
Behavior response of *Spodoptera frugiperda* caterpillars caused by deltamethrin. (**A**) Representative tracks showing the walking activity of *S. frugiperda* over a 10-min period on filter paper arenas half-impregnated with deltamethrin (upper half of each arena). Red tracks indicate high walking velocity; green tracks indicate low (initial) velocity. (**B**) Resting time and distance walked of *S. frugiperda* subjected to deltamethrin (control, LC_50_, and LC_90_ estimated values) for 10 min. Treatments (mean ± SEM) differ at *p* < 0.05 (Tukey’s mean separation test).

Representative walking tracks for *S. frugiperda* released in the half-treated arenas are observed in Figure 2A. Locomotion behavior of *S. frugiperda* caterpillars in half-treated arenas differed between the control and LCs (LC_50_ and LC_90_) of deltamethrin. The resting time was higher in the control with 358 ± 41 s, followed by LC_90_ with 224 ± 26 s, and LC_50_ with 219 ± 27 s (F_2,24_ = 4.59, *p* < 0.021). The distance traveled was higher in the control with 377 ± 44 cm, followed by LC_50_ with 256 ± 67 cm, and LC_90_ with 154 ± 39 cm (F_2,24_ = 4.53, *p* < 0.022) (Figure 2B).

### 3.4. Respiration Rate

**Figure 3:**
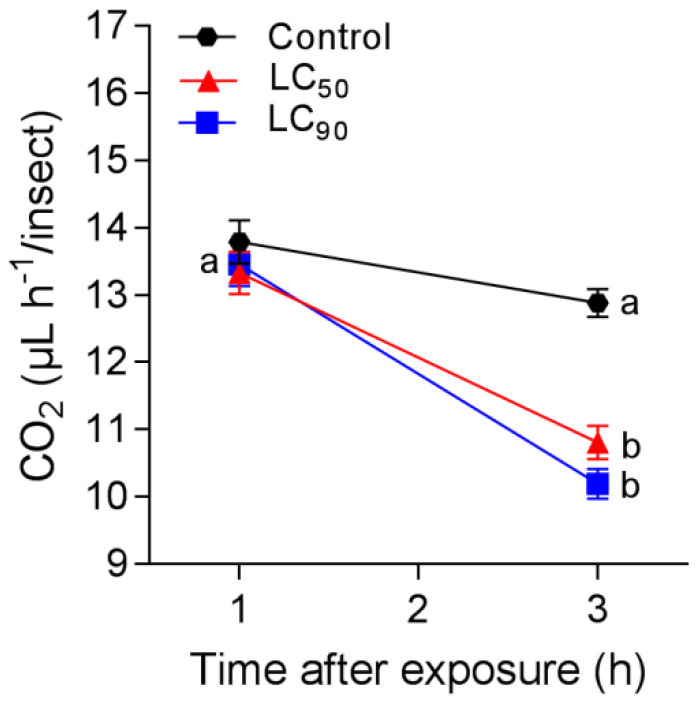
Respiration rate of *Spodoptera frugiperda* caterpillars exposed to deltamethrin (control, LC_50_, and LC_90_ estimated values) for 3 h. Treatments (mean ± SEM) differ at *p* < 0.05 (Tukey’s mean separation test).

The respiration rate of *S. frugiperda* caterpillars treated on deltamethrin began to decrease after 1 h, from 14.7 µL of CO_2_ h^−1^ in the control, to 13.5 µL of CO_2_ h^−1^ in LC_50_, and 13.1 µL of CO_2_ h^−1^ in LC_90_. After 3 h of exposure to deltamethrin, there were different respiration rates between treatments (F_2,84_ = 9.44, *p* < 0.002), exposure time (F_2,84_ = 97.4, *p* < 0.001), and treatment × time interaction (F_2,84_ = 17.1, *p* < 0.001), with 13.1 µL of CO_2_ h^−1^ in the control, 11.8 µL of CO_2_ h^−1^ in LC_50_, and 9.51 µL of CO_2_ h^−1^ in LC_90_ (Figure 3, Table 2).

### 3.5. Anti-Feeding Effect

**Figure 4:**
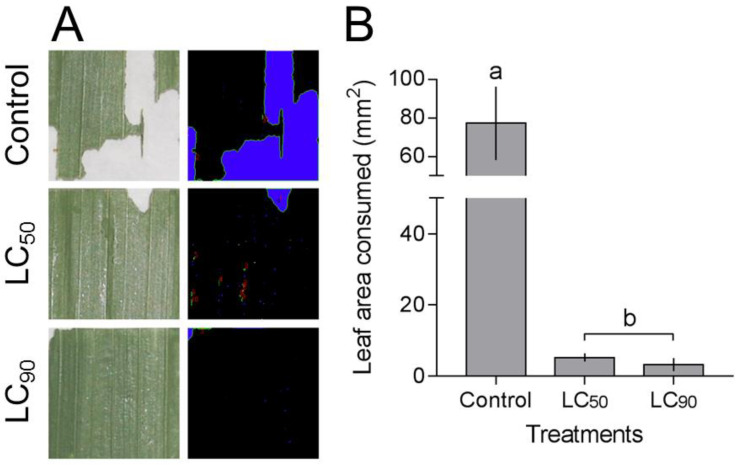
Anti-feeding effect caused by deltamethrin on *Spodoptera frugiperda* caterpillar. (**A**) Leaf section (20 × 20 mm) treated with distilled water and insecticide submitted to color binarization (black and blue) with identification and quantification of area consumed. (**B**) Leaf area consumed by *S. frugiperda* exposed to deltamethrin (control, LC_50_, and LC_90_ estimated values). Treatments (mean ± SEM) differ at *p* < 0.05 (Tukey’s mean separation test).

The leaf area consumed by *S. frugiperda* caterpillars was different in the treatments (F_2,14_ = 6.65, *p* < 0.001), being higher in the control (77.1 ± 19 mm^2^) than in those exposed to CL_90_ (5.26 ± 2 mm^2^) and CL_50_ (3.08 ± 1 mm^2^) of deltamethrin (Figure 4).

### 3.6. Histopathology

**Figure 5:**
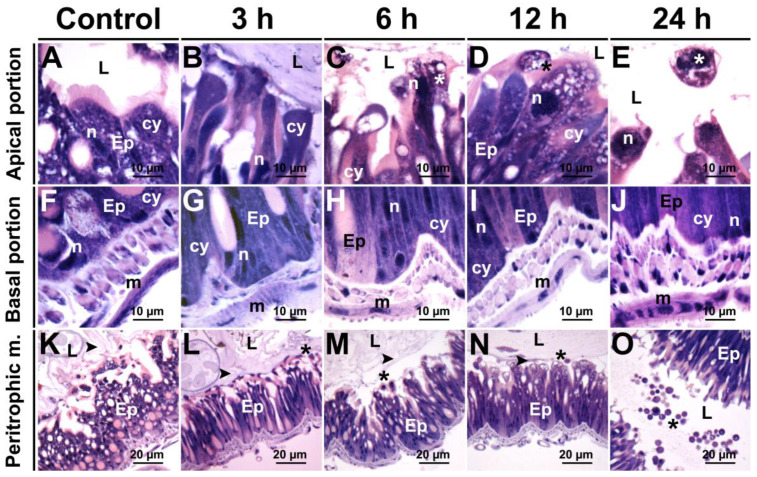
Light micrographs of the midgut of *Spodoptera frugiperda* caterpillars 3, 6, 12, and 24 h after exposure to deltamethrin. (**A**–**E**) Apical portion of digestive cells showing sequential effects with increase in vacuolization. (**F**–**J**) Basal portion of digestive cells showing sequential effects with increase in vacuolization. (**K**–**O**) Details showing sequential effects in the peritrophic matrix. Epithelium (Ep), lumen (**L**), peritrophic matrix (►), cell protrusion (*). Control (**A**,**F**,**K**).

In the control, the midgut of *S. frugiperda* had a single epithelial layer of digestive and goblet cells. Digestive cells have homogeneous cytoplasm and well-developed nuclei with predominance of decondensed chromatin. The apical surface of these cells had an evidently striated border and the midgut lumen showed a well-developed peritrophic matrix (Figure 5A,F). The basal surface of these cells was lined by muscle layers (Figure 5K). After 3 h of the insect being exposed to LC_50_ of deltamethrin, the apical surface of the midgut epithelium was irregular and the cytoplasm was highly vacuolized (Figure 5B,G,L). Protrusions of the apical epithelium towards the gut lumen, high vacuolization in the cytoplasm, and apocrine secretion were found after 6 h of insecticide exposure (Figure 5C,H,M). After 12 h exposure, there was cell vacuolization, disorganization of the striated border, cell fragmentation, nuclei with condensed chromatin, and disruption of the peritrophic matrix (Figure 5D,I,N). These features were similar to those found after 24 h of oral exposure to deltamethrin (Figure 5E,J,O). The goblet cells have a large cavity of the apical surface forming a well-developed extracellular compartment, without modifications in the insects exposed to deltamethrin (Figure 5H).

## 4. Discussion

The action of deltamethrin on *S. frugiperda* caterpillars was determined from bioassays performed in the laboratory, with an intense effect via ingestion. The insecticide caused concentration-dependent mortality of *S. frugiperda*, as reported for other insects [12,40,41]. *Spodoptera frugiperda* caterpillars exposed to LC_50_ and LC_90_ of deltamethrin changed their locomotion activity. Some individuals gradually lost mobility without signs of recovery when exposed to LC_90_. In this case, the symptoms in *S. frugiperda* agree with the effect caused by pyrethroid insecticides, which act on voltage-gated sodium channels on the axonal membrane, including those in the motor neuron [13,16]. The susceptibility to deltamethrin of other Noctuidae, such as *Chrysodeixis includens* Walker [42], *Helicoverpa armigera* Hübner [14], and *Spodoptera litura* Fabricius [43], varies according to the exposure method (contact or ingestion), but deltamethrin, in a reduced concentration (LC_50_ = 3.58 mg mL^−1^), is sufficient to cause toxicity to *S. frugiperda* by ingestion.

The high variation in *S. frugiperda* survival is mediated by the deltamethrin interaction with target sites in the nervous system, necessary to induce chronic toxicity. Time periods to induce mortality in *S. frugiperda* by this insecticide were after 80 h with LC_90_ and 120 h with LC_50_. These time differences occur because the midgut is a barrier to deltamethrin reaching the target sites. The long periods to the LCs of deltamethrin mean that *S. frugiperda* mortality obtained here may be misinterpreted as compromising the insecticide performance, since during pest outbreaks rapid pest mortality is expected [12,16], but deltamethrin inhibits the growth [40] and development [41] and interrupts the life cycle [43] of insects, and its effect against *S. frugiperda* suggests toxic effects with a possible population decrease during the first days of infestation, which is important for crop protection.

The low resting time and walked distance by *S. frugiperda* caused by deltamethrin shows its effect on the locomotion behavior of this insect, probably due to its action in the nervous system inhibiting the insect’s mobility [13,16]. The behavioral responses of insects exposed to pesticides [38,39,44] may affect substrate recognition [45], olfactory orientation[46], and foraging [41]. Changes in locomotion behavior by deltamethrin have been reported for *Anopheles harrisoni*, *Anopheles minimus* Meigan (Diptera: Culicidae) [44], *Grapholita molestans* Busk (Lepidoptera: Tortricidae) [47], *Sitophilus granarius* Linnaeus, and *Sitophilus zeamais* Motschulsky (Coleoptera: Curculionidae) [40]. Insect behavioral responses to prevent direct intoxication by insecticides include non-contact with insecticide (repellency) and evasion after brief contact (irritability) [48]. The non-preference of *S. frugiperda* walking in the half-arena treated with the insecticide suggests that the caterpillars avoid direct contact with deltamethrin.

Deltamethrin affected negatively the respiration of *S. frugiperda*, indicating physiological stress. A similar result occurs in *Anticarsia gemmatalis* Hübner (Lepidoptera: Noctuidae) exposed to azadirachtin [49], chlorpyrifos [50], and tebufenozide [26]. This decrease might be due to the low behavioral response and locomotor activity. Higher levels of walking activity are expected to result in metabolism with a high respiration rate [29,51]. The decrease in the oxygen consumption observed here may be related to the disruption of oxidative phosphorylation in respiration [49,51,52], and may result in unbalanced physiology of the insect.

The decrease in the consumption of maize leaves treated with LC_50_ and LC_90_ of deltamethrin suggests an anti-feeding effect, probably by the neurotoxic action of deltamethrin [14] causing paralysis [14,16] and, consequently, cessation of food. The rapid intoxication of *S. frugiperda*, after exposure to deltamethrin, may reduce the damage caused in plants, similar to that reported for adults of *Popillia japonica* Newman (Coleoptera: Scarabaeidae) exposed to leaves of *Tilia cordata* Mill. (Malvales: Malvaceae) treated with deltamethrin, with reduced food consumption in 96.8% [53]. Some insecticides inhibit feeding before the target insects die, and rapid action is essential for the protection of agricultural systems [11,50]. Deltamethrin and other pyrethroids reduce insect damage in maize crop as demonstrated by *Helicoverpa zea* Boddie (Lepidoptera: Noctuidae) [54] and *Ostrinia nubilalis* Hübner (Lepidoptera: Crambidae) [55].

The exposure per os to deltamethrin induces epithelial alterations in the midgut of *S. frugiperda* in short periods, although this insecticide has a neurotoxic action mode [13,21], suggesting that the midgut is a potential target organ for this insecticide. The histological changes found are characteristic of a degenerative cellular process, such as disorganization of the striated border, cytoplasm vacuolization, and cell fragmentation. In the midgut, cell degeneration is described in other insects, such as *Anticarsia gemmatalis* Hübner (Lepidoptera: Noctuidae) in response to chlorpyrifos [50] and chlorantraniliprole [51], *Apis mellifera* Linnaeus (Hymenoptera: Apidae) to iprodione [56] and spiromesifen [57], *Podisus nigrispinus* Dallas (Hemiptera: Pentatomidae) to permethrin [21] and spinosad [22], and *Aedes aegypti* (Diptera: Culicidae) to pyriproxyfen [30]. The histo-toxic effects in the midgut of *S. frugiperda* exposed to deltamethrin are mainly damages in the striated border of the digestive cells and in the peritrophic matrix. The striated border of the midgut increases the cell surface for the transport of substances [56,57,58], whereas the luminal peritrophic matrix is a physical barrier against mechanical injuries, pathogens and xenobiotic agents, and controls digestion [58,59]. The peritrophic matrix has its components (chitin and proteins) produced by the digestive cells and released by the microvilli of these cells [60,61,62]. Thus, the damage in these microvilli and in the peritrophic matrix formation may compromise the digestive process in *S. frugiperda* caterpillars.

Overall, our findings show that ingested deltamethrin has potential deleterious effects on *S. frugiperda* behavior and physiology. The application of insecticides in fields has a direct killing effect on pests exposed to lethal doses, but also results, to a certain extent, in the survival of individuals exposed to low doses over time. Some pesticides have been reported to contaminate plants from the cultivation of treated crops in the same field in previous years [63,64], so that pests may ingest lower doses of pesticides, and the changes in physiological and behavioral parameters evaluated here can be used to assess and predict the toxicity and potential efficacy of deltamethrin in the control of *S. frugiperda*.

## 5. Conclusions

This findings or this study show that deltamethrin is toxic when ingested by *S. frugiperda* caterpillars, changing their locomotion behavior, feeding, and respiration, which may be adequate to kill or interrupt the development of this pest. Thus, the findings reveal that deltamethrin histological alterations, such as disorganization of the striated border, cytoplasm vacuolization, and cell fragmentation, damage the midgut and compromise the digestive processes.

## Figures and Tables

**Table 1:** Lethal concentration of deltamethrin against *Spodoptera frugiperda* caterpillars after 5 days exposure, obtained from Probit analysis (df = 5, slope ± SE = 6.364 ± 0.53, intercept = 3.519).

No. Insects	Lethal Concentration (LC)	Estimated Concentration (mg mL^−1^)	95% Confidence Interval (mg mL^−1^)	χ^2^ (*p-*Value)
90	LC_25_	2.858	2.459–3.167	1.58(0.90)
90	LC_50_	3.588	3.251–3.909	
90	LC_75_	4.504	4.128–5.026	
90	LC_90_	5.653	5.061–6.690	

**Table 2:** Two-way ANOVA for respiration rate of *Spodoptera frugiperda* caterpillars upon exposure to lethal concentrations (LC_50_ and LC_90_) of deltamethrin for two times. DF = degrees of freedom; SS = sum of squares; MS = mean square; n = numerator; d = denominator; *p* = probability of significance; α = 0.05.

ANOVA Table	SS	DF	MS	*F* (DFn DFd)	*p*-Value
Treatments	39.31	2	19.65	*F* (2,84) = 9.44	<0.002
Time	111.9	1	111.9	*F* (1,84) = 97.4	<0.001
Treatments×time	21.68	2	10.84	*F* (2,84) = 17.1	<0.001
Residual	96.47	84	1.148		
Total	269.3	89			

## Data Availability

Data sharing not applicable.
